# Modeling Seasonal and Spatiotemporal Variation: The Example of Respiratory Prescribing

**DOI:** 10.1093/aje/kww246

**Published:** 2017-05-18

**Authors:** Eleni Sofianopoulou, Tanja Pless-Mulloli, Stephen Rushton, Peter J. Diggle

**Keywords:** chronic disease, dynamic harmonic regression, epidemiologic methods, respiratory prescribing, seasonality, spatiotemporal correlation

## Abstract

Many measures of chronic diseases, including respiratory disease, exhibit seasonal variation together with residual correlation between consecutive time periods and neighboring areas. We demonstrate a strategy for modeling data that exhibit both seasonal trend and spatiotemporal correlation, using an application to respiratory prescribing. We analyzed 55 months (2002–2006) of prescribing data from the northeast of England, in the United Kingdom. We estimated the seasonal pattern of prescribing by fitting a dynamic harmonic regression (DHR) model to salbutamol prescribing in relation to temperature. We compared the output of DHR models to static sinusoidal regression models. We used the DHR-fitted values as an offset in mixed-effects models that aimed to account for the remaining spatiotemporal variation in prescribing rates. As diagnostic checks, we assessed spatial and temporal correlation separately and jointly. Our application of a DHR model resulted in a better fit to the seasonal variation of prescribing than was obtained with a static model. After adjusting for the fitted values from the DHR model, we did not detect any remaining spatiotemporal correlation in the model's residuals. Using a DHR model and temperature data to account for the periodicity of prescribing proved to be an efficient way to capture its seasonal variation. The diagnostic procedures indicated that there was no need to model any remaining correlation explicitly.

Spatiotemporal variation in health outcomes for many chronic diseases shows periodic patterns over time (1–7) as well as more complex patterns relating to measured or unmeasured sociodemographic, behavioral, and environmental factors. When investigating health outcomes that exhibit periodic effects, the control of seasonality is a central issue; failure to do so can induce spurious correlation structure. For asthma and chronic obstructive pulmonary disease (COPD)—among the most important chronic respiratory diseases—cold air, acute respiratory infections, and pollen are known to increase the frequency and duration of symptoms. These in turn cause peaks of occurrences in winter and spring (1, 2). Several methods have been developed to control for seasonality of health outcomes, ranging from simple moving averages to more advanced smoothing techniques such as spline smoothing, kernel smoothing, or locally weighted nonparametric smoothing (8, 9). The main limitation of these smoothing methods is that they do not recognize, and therefore cannot exploit, the seasonal nature of the underlying variation. Determining the degree of smoothness can also be problematic. Harmonic regression models have been used in an attempt to provide a better approach to control for seasonal patterns when modeling respiratory and cardiovascular mortality/morbidity in relation to variations in air quality (10). Most epidemiologic studies that have taken this approach employ static versions of harmonic regression models (8, 11, 12). Dynamic models, which we present here, achieve greater flexibility by allowing the size and shape of each annual cycle to vary between years.

Dynamic harmonic regression (DHR) models have been used extensively in engineering and economics. They overcome the limitations of static models by allowing the regression coefficients associated with sine and cosine terms to vary stochastically over time (13, 14). However, DHR models have been used only rarely to model health outcomes in chronic disease epidemiology (15). Additionally, many epidemiologic studies need to link health outcomes to risk factors that vary over both space and time (16–19). Complex statistical modeling is needed to capture the temporal, spatial, and spatiotemporal correlations of health outcome data while controlling for the influence of seasonal patterns. Many health indicators of chronic diseases exhibit seasonal variation, and the increasing availability of spatiotemporally indexed data sets increases the need for flexible methodology that can account for such dependencies. In this paper, we present a flexible analysis strategy for dealing with health outcomes that vary in space and time as well as exhibiting seasonal cycles. We recommend a 2-stage strategy in which DHR modeling of seasonal trends is followed by estimation of residual spatiotemporal correlation. We demonstrate the methodology through an application to salbutamol prescribing as a proxy for exacerbations of asthma and COPD.

## METHODS

### Data

**Figure 1.**
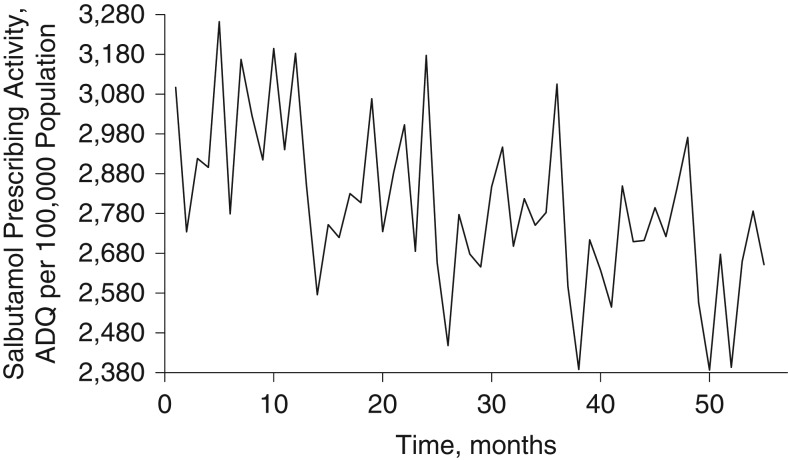
Area-wide monthly average of salbutamol prescribing activity (average daily quantities (ADQs) per 1,000 population), Northeast England, United Kingdom, 2002–2006.

Short-acting β_2_-adrenergic agonists are used to reduce asthma and COPD symptoms or to stop an acute attack in progress. For the United Kingdom in 2010, salbutamol represented 96% of short-acting β_2_-adrenergic agonist prescribing. We therefore used salbutamol prescribing as our outcome variable, interpreting this as a proxy indicator for exacerbations of asthma and COPD. This is a population-based study, and we analyzed salbutamol monthly prescribing activity in 63 primary-care practices or centers (total population >400,000 persons) in Northeast England during January 1, 2002, to July 31, 2006. We geocoded each practice location and the residential postal codes of registered patients. We then estimated the area in which 98% of registered patients were expected to live according to practice, using kernel analysis (20). We used salbutamol prescribing in units of average daily quantities, provided by the Regional Drugs and Therapeutics Center (21), which were standardized by the number of people registered with each practice, to give a measure of prescribing rate per 1,000 population monthly. We applied a log transformation to stabilize the variance and produce an approximately normal marginal distribution. Figure 1 shows the area-wide average salbutamol prescribing rate over 55 months. As expected, peaks in respiratory prescribing occurred each winter and spring, but variation from year to year was also evident, along with an overall downward trend. A map showing the average prescribing rate by practice is presented in Web Figure 1 (available at http://aje.oxfordjournals.org/).

We accessed air pollution data (for particulate matter ≤10 μm in diameter) via the National Air Quality Archive, traffic data via the local city council, and deprivation data via the Office for National Statistics. Age and sex of registered patients by practice, as well as patients’ postal codes, were accessed via the Exeter database (https://digital.nhs.uk/NHAIS/open-exeter), after obtaining ethical approval.

### Analysis

We controlled for the seasonality observed in area-wide respiratory prescribing by fitting the DHR model described below. We used harmonic components and temperature data to account for the main periodicities in the respiratory outcome that could not be explained through other area-wide covariates (i.e., respiratory infections, pollen). We then used air quality and contextual sociodemographic variables at practice level to account for the remaining spatiotemporal variation in respiratory prescribing, fitting linear mixed-effects models with the output of the DHR incorporated as an offset, described below. The construction of variables and development of candidate models have been described previously (20, 22). In this paper, we describe in detail the fitting of the final model.

### Stage 1: area-wide temporal variation

The DHR model (23) is an example of an unobserved-components model of a type that has proven to be useful for seasonal adjustment of time series data in other contexts (24, 25). The basic form of the unobserved-components model is described by Harvey (26). For our application, the model for the area-wide prescribing rate *Y(t)* was

(1) $$\begin{array}{c} Y\left(t\right)=a+\beta d\left(t\right)+\overset{k}{\underset{p=1}{\sum }}\{(A_{i,t}\cos(p\;\omega \;t)\\ \;+\;B_{i,t}\sin(p\;\omega \;t)\}+U_{t}, \end{array}$$

where *d(t)* is the average daily mean temperature in month *t* and *ω* = 2π/12 so as to give an overall 12-month cycle; see Young et al. (23). Also, *A*_*i,t*_ and *B*_*i,t*_ are stochastically varying quantities described by simple random walks, and *U*_*t*_ are independent residuals, normally distributed *N*$(0,\sigma_{u}^{2})$. The dynamic model was fitted to the data using standard maximum likelihood methods, as implemented using R (R Foundation for Statistical Computing, Vienna, Austria) package sspir (27). The DHR model can be adapted if necessary to allow spatial variation in seasonality (28).

We then used the fitted DHR models and investigated the association between salbutamol prescribing activity and lagged temperature (lags of zero, 7 days, 14 days, 21 days, and 1 month). We also repeated the model-fitting process but using static harmonic regression models, where the *A*_*it*_ and *B*_*it*_ do not vary over time, in order to compare the results with those of the corresponding dynamic models. We assessed the fit of the static and dynamic models against the observed data, both graphically and by calculating the correlation between observed data and fitted values.

### Stage 2: residual spatiotemporal variation

To assess the remaining unexplained spatiotemporal variation in salbutamol prescribing, we then fitted a linear mixed-effects model to the practice-level monthly prescribing rates, with the area-wide fitted values from stage 1 treated as an offset. Potential spatially and/or temporally varying explanatory variables at the primary-care practice level were: 1) ambient air pollution and traffic index (at different lag times); 2) income, educational, and employment deprivation; 3) average age and sex ratio of patients attending each primary-care prescribing practice, and 4) elapsed time during the study period (1, 2, … months). Ambient air pollution was the only variable for which we had no spatial information—only one monitor was located in the study area. It could therefore be included in the model at either stage 1 or stage 2. Note that air pollution may itself follow a seasonal pattern, hence area-wide air pollution is partially confounded with the area-wide seasonal time trend fitted in stage 1; we return to this point in the Discussion. The mixed-effects model was

(2) yjt=μˆt+a1(AirPollutiont)+a2(trafficjt)+a3(income)jt+a4(employmentjt)+a5(educationjt)+a6(agejt)+a7(sexjt)+a8(TimeElapsedt)+bj+εjt,

where *y*_*jt*_ is the log-transformed salbutamol prescribing rate for time (month) *t* in the primary-care center *j*, ${\hat{\mu }}_{t}$ is the population-average prescribing rate of salbutamol treated as an offset, *a* terms are static regression parameters associated with the 8 covariates, the *b*_*j*_ are practice-level random effects assumed to be normally distributed *N*$(0,\sigma_{b}^{2})$, and the ε_*jt*_ are independent residuals, also assumed to be normally distributed, *N*$(0,\sigma_{\varepsilon }^{2})$.

Next, we evaluated the fit of the final model. First, we assessed the fixed-effects part of the model by checking whether the residuals of the model met the assumptions of a multiple linear regression, namely linearity, homoscedasticity, and approximate normality. We then focused our attention on checking for stochastic dependence in space and/or time, by examining the spatial, temporal, and spatiotemporal correlation structure of the residuals. Tests to check for spatial and temporal correlation are well-developed (29–33); tests to assess spatiotemporal correlation less so (34, 35).

We assessed the temporal correlation using the autocorrelation function. This consists of a set of standard correlations calculated between a time series and its lagged version—for example, for lags 0, 1, 2, etc. Assessing spatial correlation from data recorded at an irregular set of locations is less straightforward because there is no natural direction in which to define a spatial lag. We used the empirical variogram. For a set of geostatistical data $\left(x_{i},y_{i}\right)\mathrm{: i}=1,\ldots n$, where $x_{i}$ denotes location and $y_{i}$ an associated outcome, the empirical variogram ordinates, also called semivariances, are the quantities $v_{\mathrm{ij}}=\frac{1}{2}(y_{i}-y_{j}{)}^{2}$. We plotted the $v_{\mathrm{ij}}$ against the distance $u_{\mathrm{ij}}$ within our study area (up to 20 km). Parametric models for the theoretical variogram *V(u)* can be fitted using the maximum likelihood (33). To assess the evidence for spatial correlation, we repeatedly permuted the data values among their corresponding locations in order to create a simulation envelope that indicates the amount of variation that would be expected if there is no spatial dependence.

We further examined the residuals of the model for signs of spatiotemporal correlation using the spatiotemporal extension of the variogram. This is defined as for the spatial variogram, except that empirical variogram ordinates are now considered to depend on both the spatial and temporal separations between the outcomes *y*_i_ and *y*_*j*_ (34). Our spatiotemporal data consisted of 3,465 points $x_{i},t_{i}\mathrm{: i}=1,2,\;\ldots \;3,465,$ where *x*_*i*_ denotes the location of the corresponding one of the 63 primary-care centers that prescribed respiratory medication, and *t*_*i*_ denotes month, 1, 2, … 55. The spatiotemporal variogram was computed for distances 1, 2, … 20 kilometers and time differences of 0, 1, 2, and 3 months.

Finally, we examined practice-level random effects. The random effects of the model were intended to represent the combined effects of doctors’ experience, training, and other unmeasured differences at primary-care practice level that might affect a practice's respiratory prescribing pattern. We assessed these for spatial correlation, using the empirical variogram. We also plotted 95% prediction intervals for the practice-level random effects, arranged in increasing order of their conditional mean.

## RESULTS

### Fitting seasonal variation

**Figure 2.**
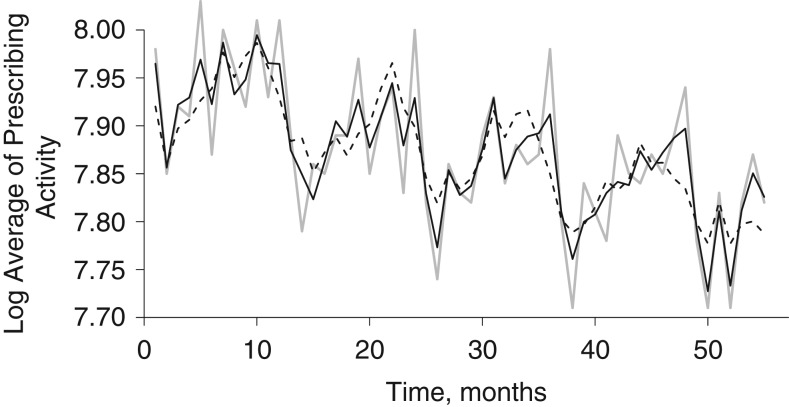
Fit of static (dashed line) and dynamic (black line) regression models for the area-wide average of salbutamol prescribing activity in relation to temperature at a 7-day lag, Northeast England, United Kingdom, 2002–2006. The observed data (gray line) is superimposed.

**Figure 3.**
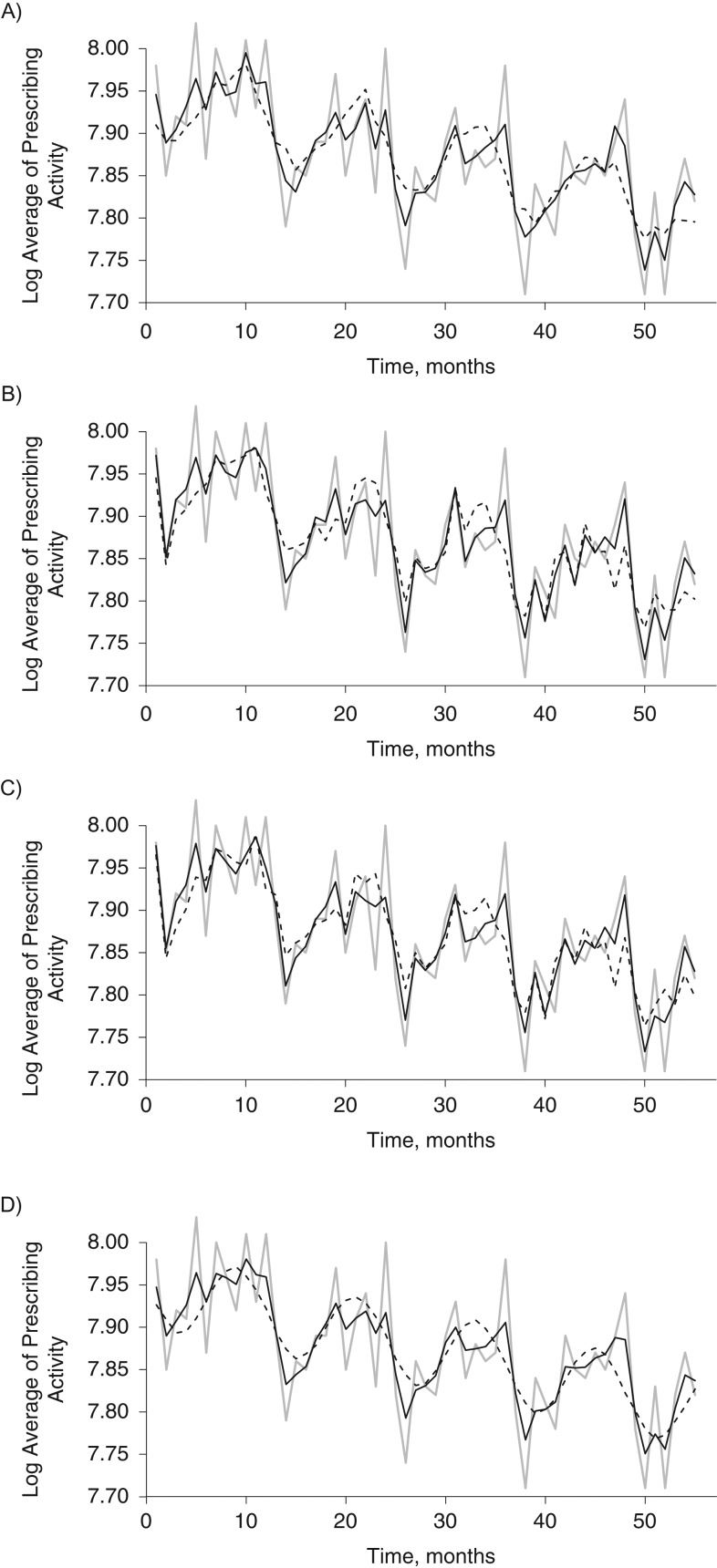
Fit of static (dashed line) and dynamic (black line) regression models for the area-wide log average of salbutamol prescribing activity in relation to temperature, Northeast of England, United Kingdom, 2002–2006. Lag of 0 days (A), 14 days (B), 21 days (C), and 1 month (D). The observed data (gray line) is superimposed.

Diagnostic plots are critical for evaluating how well an approach has dealt with seasonality or other periodic patterns. Figure 2 depicts the results of the best-fitting DHR model, which includes 7-day-lagged temperature data preceding the respiratory prescribing outcome. We also plotted the results of the static model, in order to compare the fit between the two. We evaluated several possible time lags (zero, 7 days, 14 days, 21 days, and 1 month) between area-wide average of respiratory prescribing and temperature. Figure 3 depicts the fit of the various models. Overall, the dynamic models captured the peaks and troughs better than their static counterparts.

The *R*^2^ values for the dynamic models were 0.75, 0.74, 0.71, 0.65, and 0.61 with temperature included at 7-day, 14-day, 21-day lag, zero, and 1-month lags, respectively. The *R*^2^ values for the 5 static harmonic regression models ranged from 0.46 to 0.56. The DHR that used temperature data with 7-day lag had the highest *R*^2^ value (0.75). The assumptions of linear regression were all adequately met for the dynamic model with a 7-day lag. We therefore used the predicted area-wide average of salbutamol prescribing from this model as an offset in the second-stage model.

### Fitting covariates and spatiotemporal variation

The second stage comprised a linear mixed-effects model with offset, the results of which have been described in detail previously (22). In brief, monthly averages of air pollution, income, employment deprivation, and average age of registered patients were associated with the respiratory prescribing rate, while associations with educational deprivation and local traffic flows were not statistically significant. Educational deprivation followed a different spatial pattern from that of income and employment in our study area (22), which explains why it was not being found as a significant predictor. We consider that deprivation also captured indoor air quality (i.e., smoking, occupation, housing conditions) to some extent, which was not possible to capture otherwise due to the ecological study design. In Table 1 we present the parameter estimates and standard errors of the final model. The model included general-practitioner practice-level random effects, with associated prediction intervals (Web Figure 2). The model satisfactorily captured the random effects of general practices, with small prediction intervals associated with each one. The assumptions of linear regression (normality, linearity, homoscedasticity, and absence of residual autocorrelation after adjusting for fixed and random effects) were also all adequately met (Web Figures 3–7).

**Table 1.** Associations Between Respiratory Prescribing and Covariates, From the Final Linear Mixed-Effects Model, Using Data From Northeast England, United Kingdom, 2002–2006

	Estimate	SE	*t* Value	*P* Value
Intercept	−2.060	0.141	−14.573	<0.0001
Time, months	−0.0004	0.0001	−3.301	0.001
Air pollution (PM_10_)	0.0010	0.0004	2.330	0.0198
Income deprivation	1.861	0.520	3.577	0.0003
Employment deprivation	2.287	0.810	2.824	0.0047
Age of patients	0.031	0.003	10.267	<0.0001

Abbreviation: PM_10_, particulate matter ≤10 μm in diameter; SE, standard error.

#### Spatial correlation

**Figure 4.**
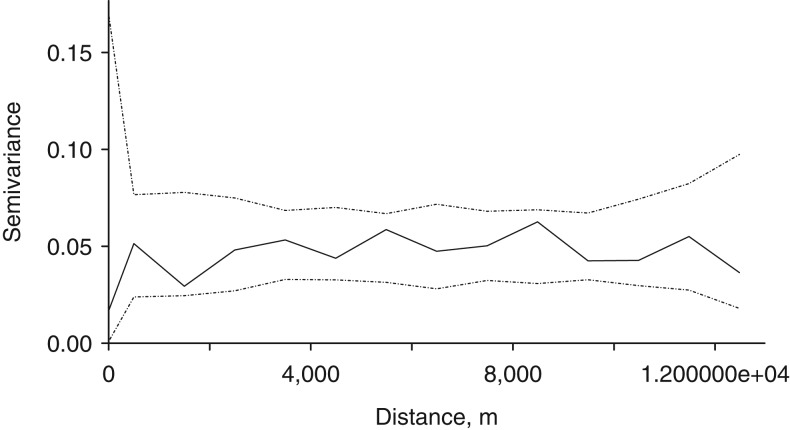
Diagnostic plot for evidence of spatial correlation of random effects. The solid line is the empirical variogram (semivariance) of the estimated random effects. The dotted lines are the pointwise upper and lower limits of empirical variograms calculated from each of 99 independent random permutations of the estimated random effects among the practice locations. The solid line lies entirely within the upper and lower limits, indicating an absence of evidence for spatial correlation.

The empirical variograms of the estimated practice-level effects from the final mixed-effects model showed an increasing trend over distances up to 8 km (Web Figure 8). In order to assess formally whether this increasing trend indicated significant residual spatial correlation, we computed envelopes for empirical variograms by permutation of the data values over the spatial locations. We computed a variogram confidence envelope from 99 independent random permutations of the residuals from a linear trend surface fitted to our data values by ordinary least squares. Figure 4 indicates no spatial correlation in random effects—the empirical variogram falls within the upper and lower limits of the simulation envelope.

#### Spatiotemporal correlation

**Figure 5.**
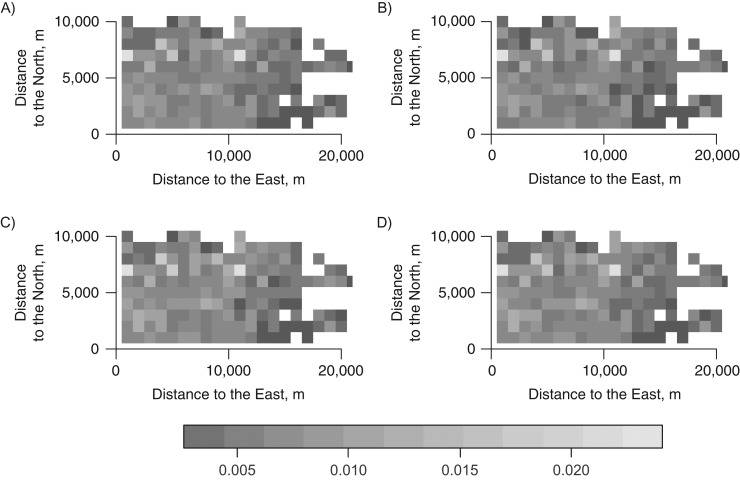
Spatiotemporal correlation diagnostic plot: spatiotemporal empirical variogram for residuals of the final model, per 1,000-m increments and time differences, showing no obvious structure, Northeast England, United Kingdom, 2002–2006. Zero months (A), 1 month (B), 2 months (C), and 3 months (D).

We further examined the residuals of the model for signs of spatiotemporal correlation via diagnostic plots. The diagnostic plot for spatiotemporal correlation of residuals is presented in Figure 5, which shows the spatiotemporal empirical variogram in the study area per 1-km increments and time differences 0–3 months. The images depicted in Figure 5 show no obvious structure and therefore support the absence of spatiotemporal correlation in the residuals. This was compared to residuals of an intercept-only model where clusters of high and low values were obvious (Web Figure 9). We conclude that the covariates captured adequately the spatiotemporal variation.

## DISCUSSION

### Main findings

We have described a flexible analytical approach for accounting for seasonality and exploring spatiotemporal residual variation using an example of respiratory prescribing. We accounted for the area-wide seasonal variation of prescribing that is influenced by respiratory infections and pollen before assessing the effect of risk factors that varied in both space and time. We focused in particular on presenting how we handled the dynamic features of the data within a relatively parsimonious modeling framework. We also presented diagnostic procedures for checking the final model assumptions. The same methodological approach could be applied to other seasonally varying health outcomes for which spatiotemporal data are available. To our knowledge, this modeling strategy has not previously been applied to health outcomes in chronic disease epidemiology, although a similar strategy has been used to model time series of black smoke concentrations (36).

### Constraints

We have used temperature as proxy for any risk factors that fluctuate with temperature. We did not have data on factors, such as respiratory infections or pollen, that would be associated with both temperature and prescribing rate. Consequently our approach of using a DHR model with temperature as a single explanatory variable provided an efficient solution to the problem of controlling for seasonality but cannot establish causality.

A related issue is that temperature itself shows seasonal variation. Consider a simplified, static version of the regression term in equation (1), namely

(3) $$\mu \left(t\right)=a+\beta d\left(t\right)+\gamma \cos\left(\frac{2\pi t}{12}\right)+\delta \sin\left(\frac{2\pi t}{12}\right).$$

Rather than include the average daily maximum temperature, *d(t)*, we could have regressed *d(t)* on seasonal sine and cosine terms to define a temperature anomaly series, d*(t), where

(4) $$d^{*}\left(t\right)=d\left(t\right)-\left(\alpha \cos\left(\frac{2\pi t}{12}\right)+b\;\sin\left(\frac{2\pi t}{12}\right)\right),$$

and fitted the regression model

(5) $$\mu^{*}\left(t\right)=a^{*}+\beta^{*}d^{*}\left(t\right)+\gamma^{*}\cos\left(\frac{2\pi t}{12}\right)+\delta^{*}\sin\left(\frac{2\pi t}{12}\right).$$

Models (3) and (4) differ in how they partition the seasonal variation between the sine-cosine and temperature terms but give identical predictions (i.e., $\mu^{⁎}\left(t\right)=\mu \left(t\right)$ for every month *t*). This emphasizes that the role of the first-stage modeling is to adjust for, rather than to explain, the area-wide seasonal variation in the health outcome of interest before, in the second stage, investigating the association of spatiotemporal covariates.

Similarly, because air pollution can vary seasonally, we could have included this variable either directly or as an air pollution anomaly series after subtraction of a seasonal trend using a second DHR fitted to the air pollution series. A potentially more serious constraint is the limitation of the source of the air pollution data to a single monitor. In reality, air pollution varies both in time and in space. If air pollution is both an important risk factor and shows substantial spatial variation, we would have expected this to show up as a spatiotemporal pattern in the residuals, but we found none. Obtaining air pollution data from a network of monitors over the study area would be a much better solution, but it was infeasible in this case.

Finally, due to the coarse time resolution (monthly) of prescribing data, it was not possible to estimate accurately the latent effects of variables related to exacerbations. However, the long latency periods that we used allowed us to check the sensitivity of the analysis as well as showing some evidence of delayed response of prescribing.

### Strengths

The dynamic modeling of seasonality allows the amplitude and phase of the seasonal cycle to vary from year to year. This is a major advantage over the models with static modeling terms that have been predominantly used in epidemiology. Secondly, the dynamic modeling approach reduces the number of parameters that are required to capture the region-wide seasonal trend and, because the model fitting is likelihood-based, the dynamic model avoids the need for any ad hoc procedure to define the degree of smoothing. Finally, the approach can easily be extended to include adjustments for other time-varying but spatially constant covariates in the first-stage model if required. For example a trend term can be added to capture an overall increase or decrease over the study period or, for an outcome variable that is recorded daily, a day-of-week effect can be included either as a smooth, cyclical curve or as a 7-level factor. In this study, we accounted for the overall falling time trend.

The 2-stage modeling approach allowed us to examine separately 2 different types of variation: region-wide temporal variation and residual spatiotemporal variation. This strategy is particularly useful for analyzing data in which there is a strong, temporally varying signal, at least part of which is not of direct interest. In this situation, using the spatially averaged, time-varying mean response from the first-stage model as an offset for the second stage, a spatiotemporal model brings 2 benefits. First, the spatiotemporal model accounts for more variation than could be achieved with separate spatial and temporal models. Second, the asymmetric separation of the total variation into temporal and spatiotemporal components, with the first taking precedence over the second, gives a more natural interpretation. Conversely, for applications in which spatial variation is dominant, essentially the same strategy could be used to partition the variation into spatial and spatiotemporal components with only technical, albeit nontrivial, changes to the first-stage modeling of temporally averaged spatial variation.

Finally, we have demonstrated the use of diagnostic procedures to examine whether periodic and spatiotemporal patterns have been adequately captured by the candidate model. The use of random-effects terms, whether to capture the overall seasonal pattern in the stage-1 analysis or to account for general-practitioner practice effects in the stage-2 analysis, controls to some degree for unmeasured predictors. Although in this study we found no evidence of significant residual spatiotemporal correlation, simply ignoring the possibility of this kind of residual can produce models that can miss potentially important sources of variation and give invalid estimates of regression-parameter standard errors.

### Implications for epidemiology and public health

We have demonstrated how using temperature data within a DHR model was able to capture the observed seasonal pattern of salbutamol prescribing. Valid data on seasonal factors—such as influenza or other respiratory infections and pollen concentrations—are usually not available, even in countries with highly organized health-care systems, so the proposed method is an efficient and pragmatic approach. In addition, the separation of purely temporal effects that can exhibit a periodic pattern from spatiotemporal effects on health outcomes can better inform health-service policies and practices, such as referral rates, blood donations, and prescribing. The proposed analyses can also add value to studies of other chronic diseases, such as cardiovascular diseases and several types of cancers, the seasonal effects of which have previously been studied using traditional methods, either by employing markers based on hospital records and deaths (4, 6, 7) or biomarkers in the context of genetic epidemiology (37–39).

Spatiotemporally indexed health data are becoming more widely available. This increased availability has been driven in part by technological advances, and it features prominently within the new discipline of health informatics. Most large health cohorts now capture the residential postal code or zip code of participants. Risk factors such as measures of deprivation, physical activity, and pollution are now also recorded in both space and time. For instance, the English Index of Multiple Deprivation, which is extensively employed by health studies, combines a range of indicators on a range of scales (the smallest including around 1,500 people) and is updated in intervening years with intercensus estimates. These resources can best be exploited fully by the parallel development of spatiotemporal statistical methods.

## Supplementary Material

Web Material

## References

[1] Global Initiative for Asthma (GINA) Global Strategy for Asthma Management and Prevention. Fontana, WI: Global Initiative for Asthma; 2016.

[2] Global Initiative for Chronic Obstructive Lung Disease (GOLD) Guide to COPD Diagnosis, Management, and Prevention. Fontana, WI: Global Initiative for Chronic Obstructive Lung Disease; 2011.

[3] Boulay F, Berthier F, Schoukroun G, et al Seasonal variations in hospital admission for deep vein thrombosis and pulmonary embolism: analysis of discharge data. BMJ. 2001;323(7313):601–602. 11557707 10.1136/bmj.323.7313.601 PMC55575

[4] Oberg AL, Ferguson JA, McIntyre LM, et al Incidence of stroke and season of the year: evidence of an association. Am J Epidemiol. 2000;152(6):558–564. 10997546 10.1093/aje/152.6.558

[5] Ashley DJ Seasonal trend in onset of lung cancer. Br Med J. 1965;1(5429):250. 10.1136/bmj.1.5429.250 PMC2165200 14228166

[6] Marti-Soler H, Gonseth S, Gubelmann C, et al Seasonal variation of overall and cardiovascular mortality: a study in 19 countries from different geographic locations. PLoS One. 2014;9(11):e113500. 25419711 10.1371/journal.pone.0113500 PMC4242652

[7] Porojnicu AC, Robsahm TE, Dahlback A, et al Seasonal and geographical variations in lung cancer prognosis in Norway. Does Vitamin D from the sun play a role. Lung Cancer. 2007;55(3):263–270. 17207891 10.1016/j.lungcan.2006.11.013

[8] Schwartz J, Spix C, Touloumi G, et al Methodological issues in studies of air pollution and daily counts of deaths or hospital admissions. J Epidemiol Community Health. 1996;50:S3–S11. 8758217 10.1136/jech.50.suppl_1.s3 PMC1060881

[9] Touloumi G, Atkinson R, Le Tertre A, et al Analysis of health outcome time series data in epidemiological studies. Environmetrics. 2004;15(2):101–117.

[10] Schwartz J Air pollution and daily mortality in Birmingham, Alabama. Am J Epidemiol. 1993;137(10):1136–1147. 8317443 10.1093/oxfordjournals.aje.a116617

[11] Hashizume M, Wagatsuma Y, Hayashi T, et al The effect of temperature on mortality in rural Bangladesh—a population-based time-series study. Int J Epidemiol. 2009;38(6):1689–1697. 19181749 10.1093/ije/dyn376

[12] von Klot S, Zanobetti A, Schwartz J Influenza epidemics, seasonality, and the effects of cold weather on cardiac mortality. Environ Health. 2012;11(1):74. 23025494 10.1186/1476-069X-11-74 PMC3517521

[13] Young PC, Pedregal DJ Recursive and en-bloc approaches to signal extraction. J Appl Stat. 1999;26(1):103–128.

[14] Kalman RE A new approach to linear filtering and prediction problems. J Basic Eng. 1960;82(1):35–45.

[15] Lundbye-Christensen S, Dethlefsen C, Gorst-Rasmussen A, et al Examining secular trends and seasonality in count data using dynamic generalized linear modelling: a new methodological approach illustrated with hospital discharge data on myocardial infarction. Eur J Epidemiol. 2009;24(5):225–230. 19288215 10.1007/s10654-009-9325-z

[16] Tolbert PE, Mulholland JA, MacIntosh DL, et al Air quality and pediatric emergency room visits for asthma in Atlanta, Georgia, USA. Am J Epidemiol. 2000;151(8):798–810. 10965977 10.1093/oxfordjournals.aje.a010280

[17] Maas J, Verheij RA, Groenewegen PP, et al Green space, urbanity, and health: how strong is the relation. J Epidemiol Community Health. 2006;60(7):587–592. 16790830 10.1136/jech.2005.043125 PMC2566234

[18] Jerrett M, Burnett RT, Ma R, et al Spatial analysis of air pollution and mortality in Los Angeles. Epidemiology. 2005;16(6):727–736. 16222161 10.1097/01.ede.0000181630.15826.7d

[19] Diez-Roux AV, Nieto FJ, Muntaner C, et al Neighborhood environments and coronary heart disease: a multilevel analysis. Am J Epidemiol.1997;146(1):48–63. 9215223 10.1093/oxfordjournals.aje.a009191

[20] Sofianopoulou E, Rushton S, Rubin G, et al Defining GP practice areas based on true service utilisation. Health Place. 2012;18(6):1248–1254. 23041911 10.1016/j.healthplace.2012.08.006

[21] Walley T, Roberts D Average daily quantities: a tool for measuring prescribing volume in England. Pharmacoepidemiol Drug Saf. 2000;9(1):55–58. 19025803 10.1002/(SICI)1099-1557(200001/02)9:1<55::AID-PDS467>3.0.CO;2-H

[22] Sofianopoulou E, Rushton SP, Diggle PJ, et al Association between respiratory prescribing, air pollution and deprivation, in primary health care. J Public Health (Oxf). 2013;35(4):502–509. 24293452 10.1093/pubmed/fdt107

[23] Young PC, Pedregal DJ, Tych W Dynamic harmonic regression. J Forecast. 1999;18(6):369–394.

[24] Gersch W, Kitagawa G The prediction of time series with trends and seasonalities. J Bus Econ Stat. 1983;1(3):253–264.

[25] Harvey A, Scott A Seasonality in dynamic regression models. Econ J. 1994;104(427):1324–1345.

[26] Harvey AC Forecasting, Structural Time Series Models and the Kalman Filter. Cambridge, UK: Cambridge University Press; 1989.

[27] Dethlefsen C, Lundbye-Christensen S Formulating state space models in R with focus on longitudinal regression models. J Stat Softw. 2006;16(1):1–15.

[28] Romanowicz R, Young P, Brown P, et al A recursive estimation approach to the spatio-temporal analysis and modelling of air quality data. Environ Model Softw. 2006;21(6):759–769.

[29] Cressie N Statistics for Spatial Data. Revised ed New York, NY: Wiley; 1991.

[30] Diggle PJ Statistical Analysis of Spatial Point Patterns. 2nd ed London, UK: Arnold; 2003.

[31] Daley DJ, Vere-Jones D An Introduction to the Theory of Point Processes. 2nd ed New York, NY: Springer; 2003.

[32] Cox DR, Isham V Point Processes. London: Chapman and Hall; 1980.

[33] Diggle PJ, Ribeiro PJ Model-Based Geostatistics. New York: Springer; 2007.

[34] Gabriel E, Diggle PJ Second-order analysis of inhomogeneous spatio-temporal point process data. Stat Neerl. 2012;66(4):472–491.

[35] Pebesma E spacetime: spatio-temporal data in R. J Stat Softw. 2012;15(7):1–7.

[36] Fanshawe TR, Diggle PJ, Rushton S, et al Modelling spatio-temporal variation in exposure to particulate matter: a two-stage approach. Environmetrics. 2008;19(6):549–566.

[37] De Jong S, Neeleman M, Luykx JJ, et al Seasonal changes in gene expression represent cell-type composition in whole blood. Hum Mol Genet. 2014;23(10):2721–2728. 24399446 10.1093/hmg/ddt665 PMC3990170

[38] Dopico XC, Evangelou M, Ferreira RC, et al Widespread seasonal gene expression reveals annual differences in human immunity and physiology. Nat Commun. 2015;6:7000. 25965853 10.1038/ncomms8000 PMC4432600

[39] Ricceri F, Trevisan M, Fiano V, et al Seasonality modifies methylation profiles in healthy people. PLoS One. 2014;9(9):e106846. 25210735 10.1371/journal.pone.0106846 PMC4161384

