# Supplementary material for: Modeling Seasonal and Spatiotemporal Variation: The Example of Respiratory Prescribing
Source: Am J Epidemiol. 2017 May 18;186(1):101–8. doi: 10.1093/aje/kww246 (PMC5860516; doi:10.1093/aje/kww246)

Web Figure 1 Average prescribing rate by practice, in the study area

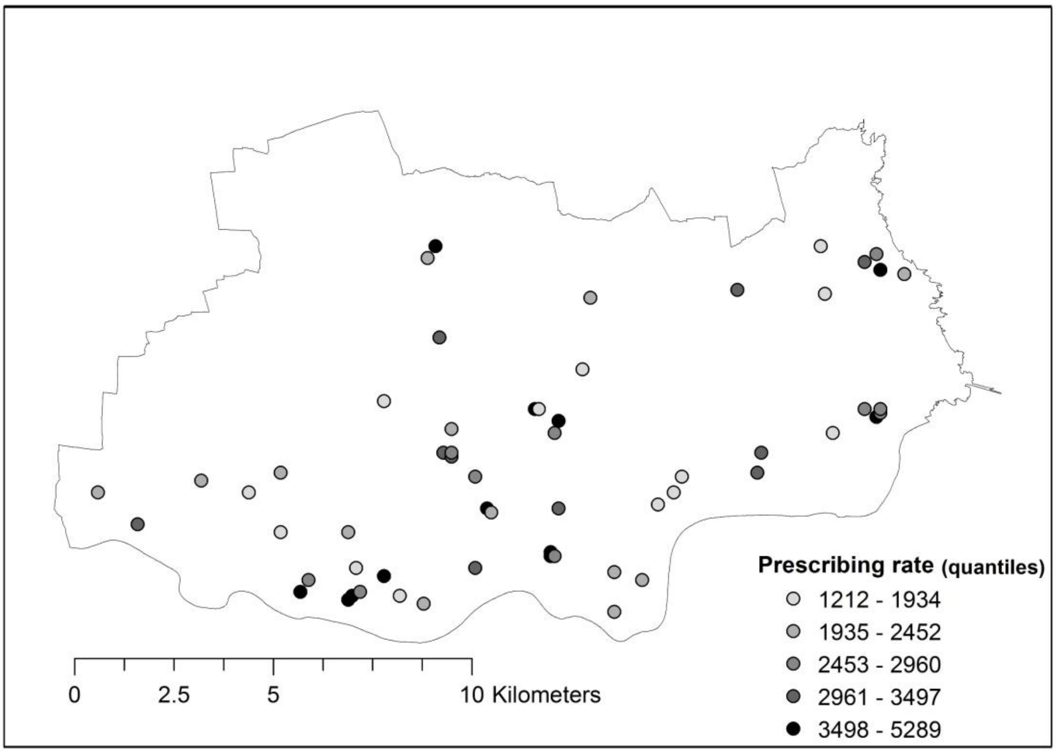

Web Figure 2 Plotting the random effects in the form of 95% prediction intervals.

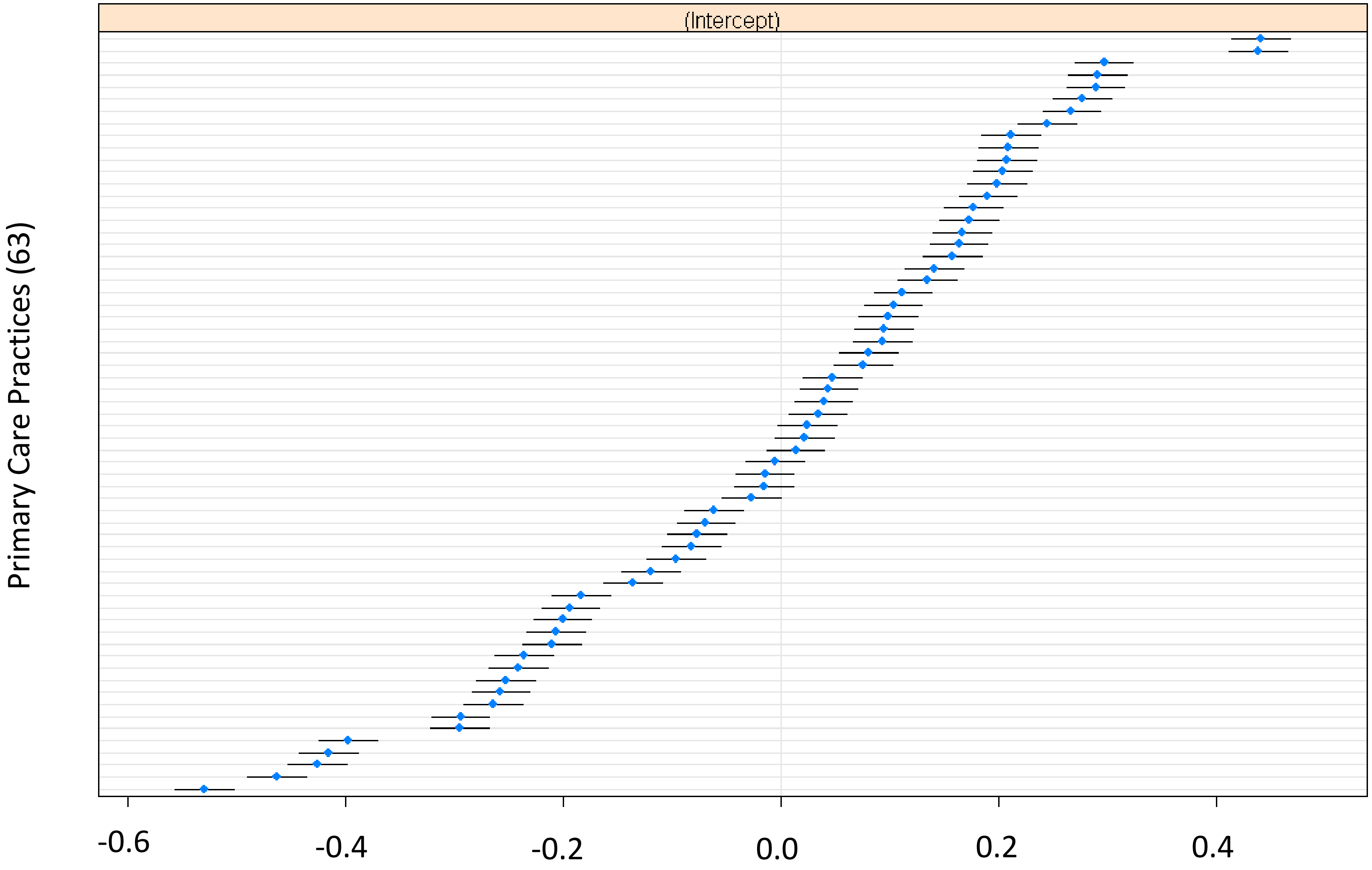

Web Figure 3 - 7: Diagnostics for the final mixed effects model

Web Figure 3 Normality probability plot of the residuals.

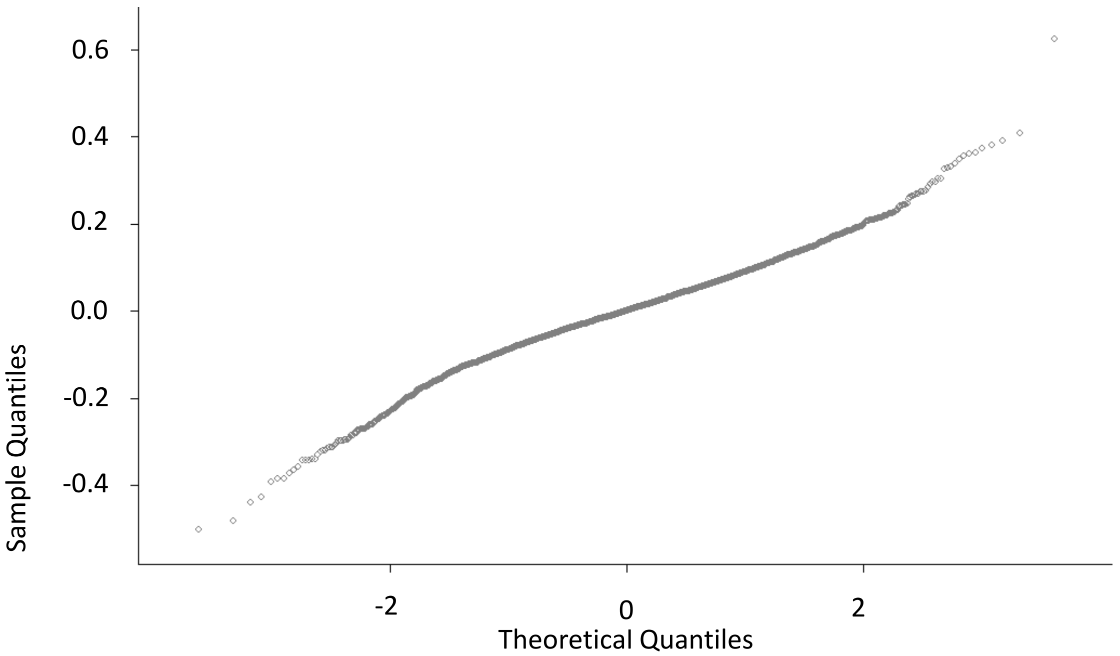

Web Figure 4 Linearity: The observed values plotted against the fitted values.

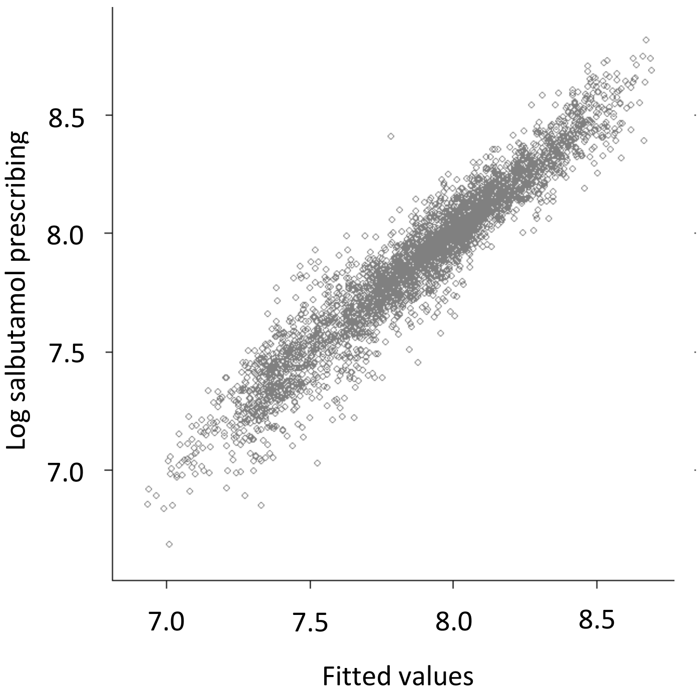

Web Figure 5 Homoscedasticity & Linearity: The residuals of the final model plotted against the fitted values.

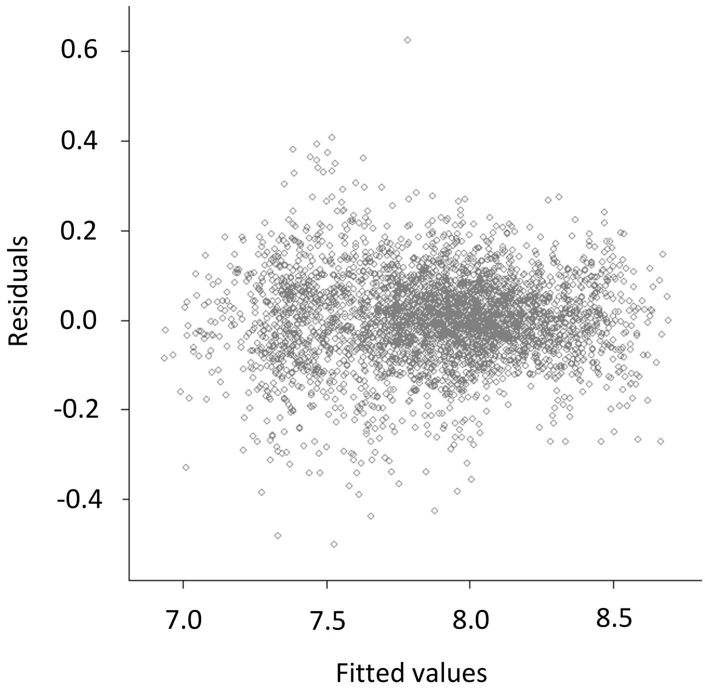

Web Figure 6 Homoscedasticity: The residuals of the final model plotted against time.

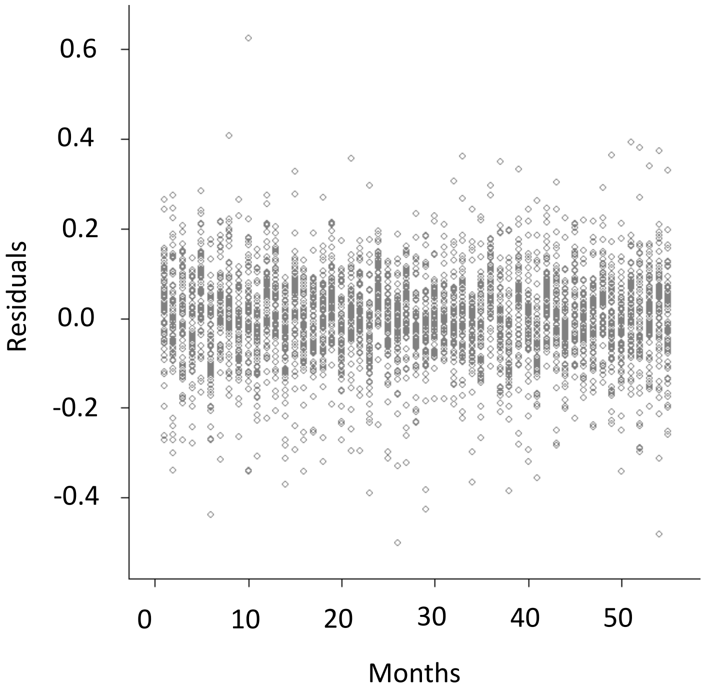

Web Figure 7 Temporal interdependence of residuals estimated by the autocorrelation function.

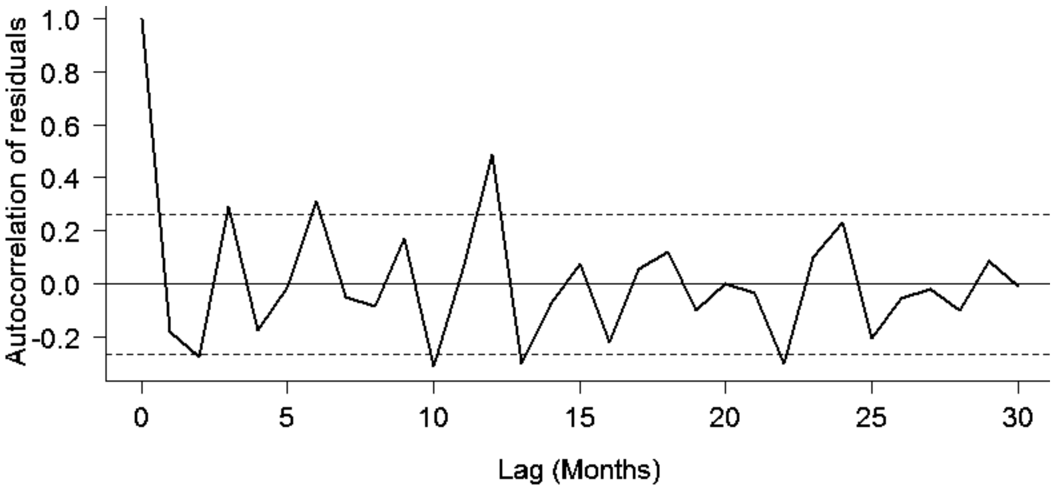

Web Figure 8 Empirical variogram in random effects of the final mixed effects model.

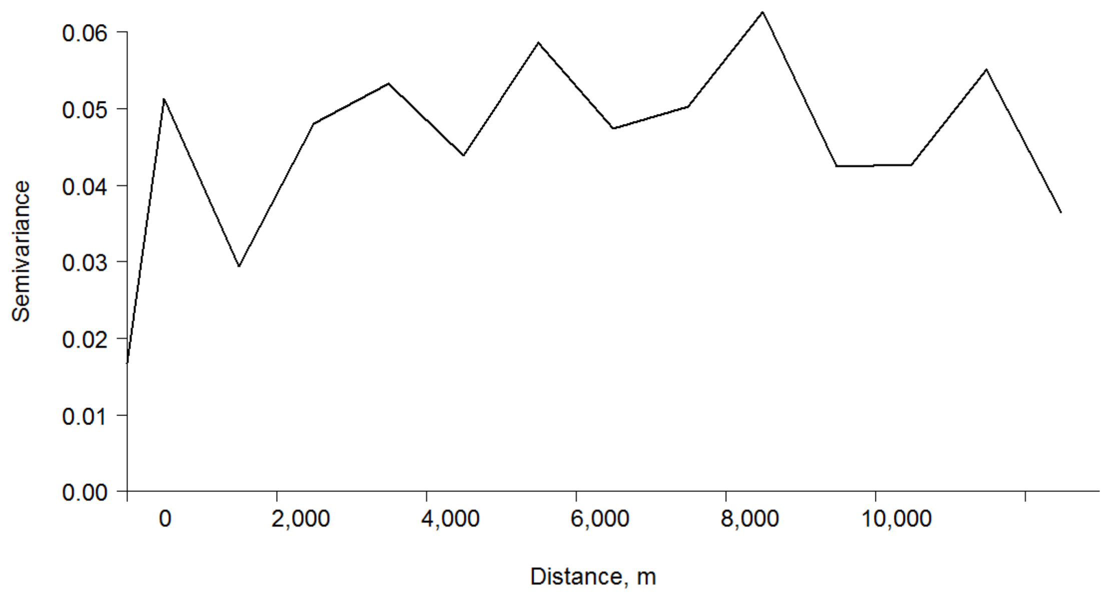

Web Figure 9 Spatio-temporal correlation diagnostic plot: Spatio-temporal empirical variogram for residuals of the intercept only model, per 1000m increments and time-differences at: a) 0 months, b) 1 month, c) 2 months and d) 3 months.

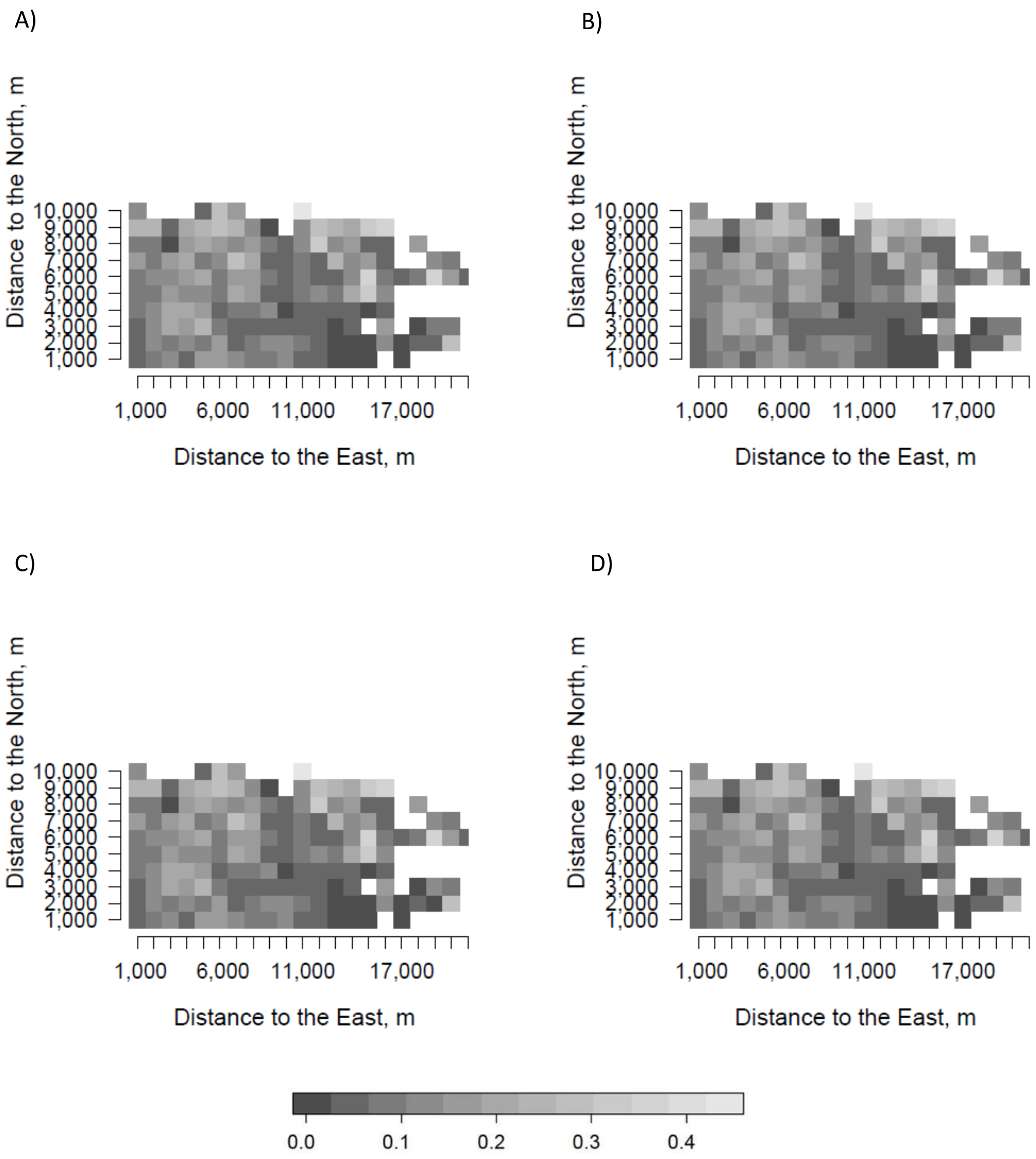

Supplement: Web Material [file kww246sofianopoulouwebmaterialfinal.pdf]
